# Surfactants’ Interplay with Biofilm Development in *Staphylococcus* and *Candida*

**DOI:** 10.3390/pharmaceutics16050657

**Published:** 2024-05-15

**Authors:** Florin Aonofriesei

**Affiliations:** Department of Natural Sciences, Faculty of Natural and Agricultural Sciences, Ovidius University of Constanta, 1, University Street, 900470 Constanța, Romania; aonofriesei_florin@yahoo.fr

**Keywords:** *Staphylococcus*, *Candida*, surfactants, microbial biofilm

## Abstract

The capacity of micro-organisms to form biofilms is a pervasive trait in the microbial realm. For pathogens, biofilm formation serves as a virulence factor facilitating successful host colonization. Simultaneously, infections stemming from biofilm-forming micro-organisms pose significant treatment challenges due to their heightened resistance to antimicrobial agents. Hence, the quest for active compounds capable of impeding microbial biofilm development stands as a pivotal pursuit in biomedical research. This study presents findings concerning the impact of three surfactants, namely, polysorbate 20 (T20), polysorbate 80 (T80), and sodium dodecyl sulfate (SDS), on the initial stage of biofilm development in both *Staphylococcus aureus* and *Candida dubliniensis.* In contrast to previous investigations, we conducted a comparative assessment of the biofilm development capacity of these two taxonomically distant groups, predicated on their shared ability to reduce TTC. The common metabolic trait shared by *S. aureus* and *C. dubliniensis* in reducing TTC to formazan facilitated a simultaneous evaluation of biofilm development under the influence of surfactants across both groups. Our results revealed that surfactants could impede the development of biofilms in both species by disrupting the initial cell attachment step. The observed effect was contingent upon the concentration and type of compound, with a higher inhibition observed in culture media supplemented with SDS. At maximum concentrations (5%), T20 and T80 significantly curtailed the formation and viability of *S. aureus* and *C. dubliniensis* biofilms. Specifically, T20 inhibited biofilm development by 75.36% in *S. aureus* and 71.18% in *C. dubliniensis*, while T80 exhibited a slightly lower inhibitory effect, with values ranging between 66.68% (*C. dubliniensis*) and 65.54% (*S. aureus*) compared to the controls. Incorporating these two non-toxic surfactants into pharmaceutical formulations could potentially enhance the inhibitory efficacy of selected antimicrobial agents, particularly in external topical applications.

## 1. Introduction

Biofilms constitute a significant adaptive strategy within the microbial realm [1]. They arise from cell attachment to surfaces, followed by encapsulation within a complex matrix primarily composed of polysaccharides, proteins, and inorganic compounds [2]. The growth of biofilms confers numerous advantages to micro-organisms compared to planktonic cells [3], including (i) the enhanced access to nutrients [1]; (ii) a heightened stability under adverse conditions; and (iii) an increased resistance to biocidal agents, among others [4,5]. Biofilms can encompass single or multiple microbial species, with cells adhering to both biotic and abiotic surfaces. The formation of microbial biofilms is a multifaceted process involving several sequential stages: (i) reversible cell attachment; (ii) irreversible attachment; (iii) biofilm maturation; and (iv) biofilm detachment or dispersion. Microbes have the capacity to colonize and develop biofilms on a wide array of surfaces, whether natural or artificial [6]. Their proliferation can lead to various adverse effects, particularly concerning medical instruments. When biofilms form on indwelling devices such as catheters, prosthetic heart valves, pacemakers, or contact lenses, they can instigate challenging-to-treat infections [7]. While numerous micro-organisms are potentially capable of biofilm formation, prevalent cases are often associated with genera such as *Staphylococcus*, *Pseudomonas*, and *Candida* [2]. The antimicrobial resistance exhibited by biofilm-producing pathogens is multifaceted and intricate. One pivotal factor is the limited penetration of antimicrobial agents into the biofilm matrix. The structure of biofilms impedes the diffusion of antimicrobials, a phenomenon that varies depending on factors such as the species of the pathogen, the antimicrobial agent employed, and the growth stage of the biofilm [8,9,10,11]. Another critical aspect of resistance is the slowed growth rate resulting from specific conditions within the biofilm matrix, such as hypoxia [10]. Quorum sensing can also play a significant role in bolstering the resistance of the cell population within the biofilm to antimicrobial action [11]. Additionally, the presence of exopolysaccharides and certain enzymes that alter the composition of antibiotics within the biofilm matrix further enhances the antimicrobial resistance of biofilm-forming pathogens [12]. A common trait in the pathogenesis of *Candida* species is their capacity to form biofilms, which confer protection against the host’s immune system and antifungal drugs alike. Biofilms augment pathogen colonization and the incidence of systemic or superficial infections, particularly in immunocompromised patients [13,14]. It is estimated that over 60% of chronic infections are attributable to micro-organisms capable of biofilm production [15]. Infections involving biofilm-forming pathogenic micro-organisms pose formidable treatment challenges due to their heightened antimicrobial resistance. The available data suggest that antibiotic resistance contributes to an estimated 600–700,000 deaths annually [16]. These infections pose significant therapeutic challenges, primarily due to the high resistance of involved micro-organisms to antifungal agents [17]. *Candida* species frequently cause severe infections associated with elevated mortality rates [18,19]. Each *Candida* species exhibits differences in biofilm formation ability and antifungal resistance profiles. Given the resistance conferred by these biofilm-associated infections, there is an urgent imperative to control biofilm development and identify more effective therapeutic interventions [20]. Identifying these interventions entails studying the virulence factors [21] of these pathogens, among which the capacity to form biofilms is paramount. *Candida dubliniensis* was initially described by Sullivan et al. [22], exhibiting phenotypic traits closely resembling those of *C. albicans*. However, it possesses several phenotypic and molecular characteristics that distinguish it from the latter. *C. dubliniensis* is less frequently isolated from clinical specimens and exhibits a lower tolerance to environmental stress compared to *C. albicans* [23,24]. Unlike *C. albicans*, *C. dubliniensis* can reduce 2,3,5-triphenyl-2H-tetrazolium chloride (TTC) to 1,3,5-triphenyltetrazolium formazan (TPF, formazan) [25]. This capacity shared with many pathogenic biofilm-producing bacteria enabled simultaneous comparisons between taxonomically and ultrastructurally distant groups. Key factors influencing biofilm development include [19]: (i) substrate attachment quality; (ii) available carbon sources; and (iii) intrinsic genetic factors specific to individual micro-organisms. As elucidated above, a fundamental step in biofilm formation is cell adhesion to a substrate, followed by discrete colony growth [26]. Cell adhesion to diverse surfaces can be influenced by various physical, chemical, and biological factors. Recent studies have identified several potential avenues, albeit in the laboratory stage, to combat the antibiotic resistance of biofilm-producing pathogens, including (i) matrix-degrading enzymes like dispersin B [27]; (ii) quorum sensing inhibitors [28]; and (iii) surface coatings [29,30]. Surfactants are molecules capable of reducing surface tension at interfaces such as air/liquid or liquid/solid, leading to the formation of amphipathic micelles [31]. Consequently, compounds with surfactant properties can impede cell settlement on different surfaces and the subsequent development of microbial biofilms [32,33,34]. Moreover, surfactants can influence various structures (cell wall or cell membrane) or microbial physiological functions (e.g., active molecule transfer from the environment) [35,36]. Surfactant properties hold particular promise in the biomedical realm for controlling infections caused by biofilm-forming pathogens [37,38]. With this in mind, this study aims to assess the impact of a range of ionic and non-ionic surfactants on biofilm production in *Staphylococcus* and *Candida*. Polysorbate 20 (T20), polysorbate 80 (T80), and sodium dodecyl sulfate (SDS) were employed at various concentrations to evaluate biofilm development in clinical and reference strains of Staphylococcus aureus and *Candida dubliniensis*. Biofilm production levels were estimated using a tetrazolium salt reduction assay to measure living cells remaining in the biofilm after removal of free-floating cells.

## 2. Materials and Methods

*Strains and Cultivation*. To investigate the impact of surfactants (polysorbate 20, polysorbate 80, and sodium dodecyl sulfate), we utilized eight strains derived from *Staphylococcus* and *Candida*, encompassing both reference and clinical strains (Table 1). Reference strains were procured from Microbiologics (St. Cloud, MN, USA), while clinical strains were graciously provided by a medical microbiology laboratory affiliated with Ovidius University of Constanta, Romania. Clinical specimens from skin infections were cultured on Mannitol Salt Agar (MSA, Liofilchem, Italy), with pure cultures identified as S. *aureus* based on morphological characteristics, Gram staining, coagulase and catalase tests, and DNase assays. Clinical strains of *C. dubliniensis* were isolated from oral infections and identified through chlamydospore formation, growth at 42 °C tests, and carbohydrate assimilation using the API 20C Aux system [37]. The susceptibility of clinical strains from both species to a broad spectrum of antibiotics and antifungals was assessed (Table 1).

*Growth and Maintenance of Staphylococcus*. Staphylococcus cultures were maintained prior to experimentation on Mannitol Salt Agar (MSA, Liofilchem), while *Candida* cultures were subcultured on Sabouraud Dextrose Agar (SDA, Oxoid).

*Staphylococcus Biofilm*. To evaluate biofilm development, we followed a modified version of the method described by Sabaeifard et al. [39] and Brown et al. [40]. This method relies on the metabolic activity of cells to reduce a tetrazolium salt (2,3,5-triphenyltetrazolium chloride—TTC) to red formazan (triphenylformazan—TPF). The extracted TPF is then quantified spectrophotometrically.

**Figure 1:**
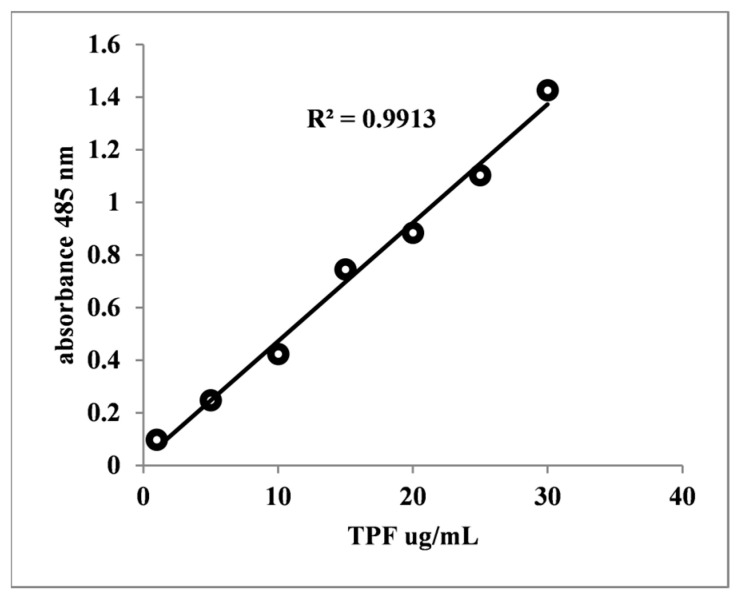
Calibration curve illustrating the correlation between absorbance at 485 nm and formazan concentration.

Before experimentation, *Staphylococcus* stock cultures were subcultured on Tryptone Soy Agar (TSA, Sigma Aldrich, St. Louis, MO, USA). Subsequently, they were inoculated into Tryptone Soy Broth (TSB, Thermo Scientific, Waltham, MA, USA) and incubated overnight. Following incubation, the cultures (6–7 × 10^6^ CFU/mL) were diluted 1:100, and 100 µL of the diluted culture was inoculated into test tubes containing TSB supplemented with 2% glucose and surfactants (T20, T80) at final concentrations of 1%, 2%, and 5% (*w*/*v*), except for SDS, where the concentration varied from 0.1 to 0.5% (*w*/*v*). Each experimental condition was inoculated in triplicate. The positive control received 100 µL of the inoculum, while the negative control consisted of sterile TSB. The test tubes were then aerobically incubated for 48 h at 37 °C. After the incubation period, planktonic cells were removed, and the tubes were washed four times with sterile phosphate-buffered saline (PBS) (pH = 7.4) to eliminate all free-floating cells. Subsequently, the tubes received 600 µL of ¼-strength Ringer solution, 300 µL of sterile PBS, 90 µL of 5% glucose, and 10 µL of 1% TTC solution. The tubes were further incubated for 24 h to allow for the reduction of TTC to TPF by living cells present in the biofilm matrix. At the end of the incubation period, the tubes were centrifuged (8000/min), the supernatant was removed, and TPF was extracted using absolute methanol (Ridl de Haen, Seelze, Germany) three to four times. Samples were then read using a double-beam Jasco UV–Vis spectrophotometer at 485 nm. The amount of TPF produced by the biofilm was quantified using a calibrated curve ranging from 1 µg to 30 µg TPF (Figure 1).

*Candida Biofilm*. Sabouraud Dextrose Agar (SDA) was utilized for subculturing *Candida* strains before experimentation. The effect of surfactants on the attachment step was assessed by measuring biofilm metabolic activity using the TTC reduction assay. We employed a slightly modified protocol based on the methods described by Paramanantham et al. [41]. Cultures of *Candida dubliniensis* were diluted in Sabouraud Dextrose Broth (SDB, Oxoid, Basingstoke, UK) to achieve a density of 1 × 10^6^ cells/mL, which were then transferred to test tubes containing varying concentrations of surfactants ranging from 1 to 5%. The suspensions were incubated for 48 h at 35 °C. Subsequently, planktonic cells were removed by washing the tubes three times, and then 600 µL of Ringer solution, 300 µL of PBS, 90 µL of 5% glucose, and 10 µL of 1% TTC were added to each tube. The tubes were incubated again for 24 h at 35 °C. After incubation, the tubes were centrifuged, the supernatant was removed, and the TPF formed was extracted using methanol. Absorbance was determined spectrophotometrically at 485 nm.

*Relationship Between Cellular Density and TTC Reduction.* To evaluate the quantitative relationship between cell density and the intensity of TTC reduction, overnight cultures were harvested by centrifugation and diluted two-fold in series of test tubes until no visual turbidity was observed. Dilutions were made in sterile Ringer solution (Merck, Lowe, NJ, USA) supplemented with 10 µL of 1% TTC, 10 µL of 10% glucose solution, and 100 µL of phosphate buffer (pH = 7.4). The test tubes were then incubated at 35–36 °C for 8 h, followed by centrifugation, removal of the supernatant, and extraction of TPF three to four times using absolute methanol (Ridl de Haen). The concentration of TPF was determined spectrophotometrically at 485 nm using a Jasco UV–Vis spectrophotometer.

*Planktonic Growth* vs. *Attached Growth*. To assess the ability of strains to attach to the walls of 2 mL test tubes (Eppendorf), we evaluated both planktonic and biofilm growth. The culture was removed, centrifuged, and incubated with TTC, glucose, and phosphate buffer for 24 h. After removing the cultures, the tubes were washed three to four times with sterile Ringer solution to eliminate all planktonic cells. Subsequently, the tubes were filled with Ringer solution (600 µL), phosphate buffer (300 µL), TTC (10 µL), and glucose (10 µL), and incubated for 24 h at 37 °C. After incubation and TTC reduction, the tubes were centrifuged, the supernatant was removed, and TPF was extracted and quantified as previously described.

*Effect of Surfactants on Attached Growth*. To facilitate biofilm development, overnight cultures of *Staphylococcus* were inoculated (10 µL) into TSB supplemented with 2% glucose. For *Candida* strains, inoculation was performed in SDB supplemented with 2% glucose. The cultures were incubated for 48 h at 37 °C. After washing and removing planktonic cells, the test tubes (Eppendorf 2 mL safe-lock tubes) were filled with Ringer solution (600 µL), phosphate buffer (300 µL), TTC (10 µL), and glucose (10 µL). Sterile solutions of T20 and T80 were added to reach final concentrations of 1%, 2%, and 5%, while SDS was added to achieve concentrations of 0.1%, 0.2%, and 0.5%. The tubes were then incubated for 24 h at 37 °C. After incubation, the tubes were centrifuged, the supernatant was removed, and TPF was extracted and quantified as described above. Statistical analysis of the data was performed using Pearson product–moment correlation coefficients and Student’s *t*-test. A paired *t*-test was utilized to compare the degree of biofilm development between the controls and experimental variants with different concentrations of surfactants. Specifically, the *t*-tests aimed to highlight differences between the controls and experimental variants, representing the null hypothesis (H_0_ when the difference between controls and experimental variants is 0) versus the alternative hypothesis (H_a_—indicating that values differ between the two groups). Pearson correlations were employed to define the relationship between TTC reduction in biofilms versus planktonic cultures. A positive correlation indicates similar metabolic activity and likely similar cell densities in biofilms compared to planktonic cultures. Positive correlation implies that the two variables (TTC reduction in planktonic cultures versus TTC reduction in biofilms) vary in the same direction and are more or less similar in metabolic activity. The data were processed and analyzed using the STW Statistics 18 software package.

## 3. Results

*Cellular Density and TTC Reduction Relationship*. Determining the biofilm viability relies on living cells’ capacity to reduce TTC, yielding red TPF. The TTC reduction intensity varies based on parameters such as the culture medium composition, temperature, inhibitors, and oxygen presence.

Variability exists even within the same genus or strains of a microbial species. As previously mentioned, biofilms consist of microbial cells on a solid surface, embedded in a polysaccharide matrix, varying in thickness and cell count.

**Figure 2:**
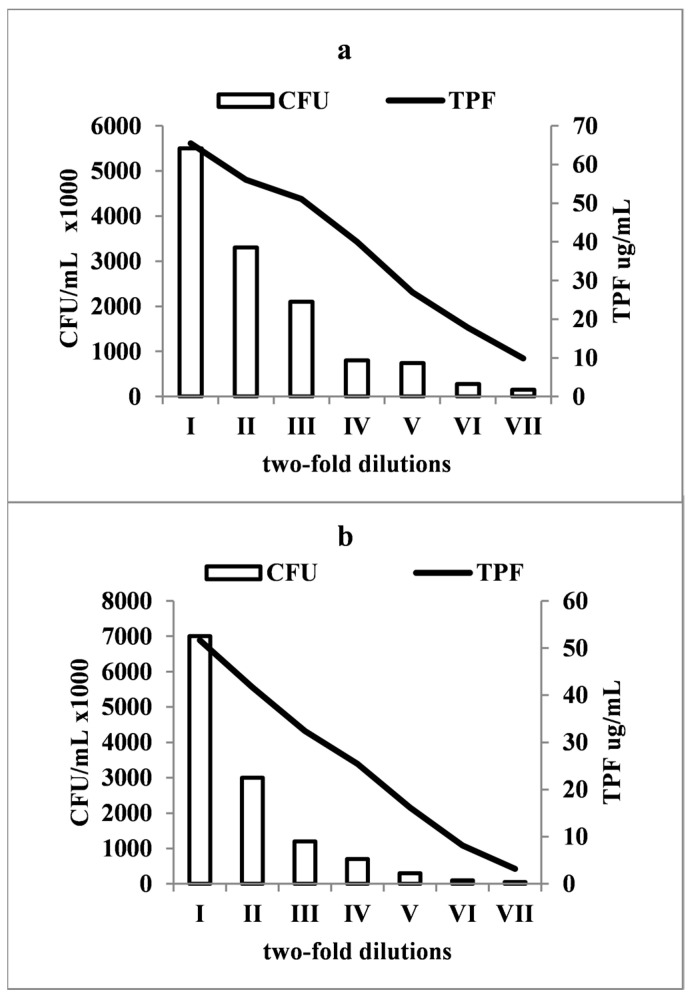
(**a**,**b**). The relationship between cell density and TPF production in planktonic cultures of S. aureus SaCS3 (**a**) and C. *dubliniensis* CdCS1 (**b**). A dense inoculum between 6 × 10^6^ CFU/mL (*S. aureus* SaCS3) and 7 × 10^6^ CFU/mL (*C. dubliniensis* CdCS1) was diluted two-fold until no visible turbidity was observed. Dilutions were prepared in two experimental setups: (**a**) 1 mL of culture was diluted two-fold in MHB (*S. aureus* SaCS3) and SDB (*C. dubliniensis* CdCS1). The samples underwent a further 1/10 dilution, with 100 µL extracted and inoculated onto MHA (*S. aureus* SaCS3) and SDA (*C. dubliniensis* CdCS1). Petri dishes in triplicate were incubated, colonies counted, and density expressed as CFU/mL. (**b**) 1 mL of each strain was diluted two-fold in Ringer’s solution, followed by glucose and TTC addition. Cultures were incubated, centrifuged, TPF extracted, and quantified spectrophotometrically.

To estimate the cell count from a biofilm constitution, a calibration correlating the TTC reduction capacity with a viable cell count was conducted. Dilutions from 24 h cultures in Ringer’s solution for both *Staphylococcus* and *Candida* were prepared. The living cell count estimation involved a further dilution of the initial two-fold dilutions, with 100 µL inoculated onto SDA (*Candida*) and TSA (*Staphylococcus*), followed by incubation and CFU counting. To quantify the TPF production and correlate it with the cell density, similar two-fold dilutions from overnight cultures were supplemented with TTC and glucose and incubated under the same conditions. After incubation, the resulting TPF was extracted and quantified spectrophotometrically. The results demonstrated a close correlation between the cell density and TTC reduction (Figure 2a,b). Positive correlations were observed in both cases, *Staphylococcus* (r = 0.89) and *Candida* (r = 0.88), indicating that the reduction intensity can indicate cell numbers within certain limits in the biofilm.

**Figure 3:**
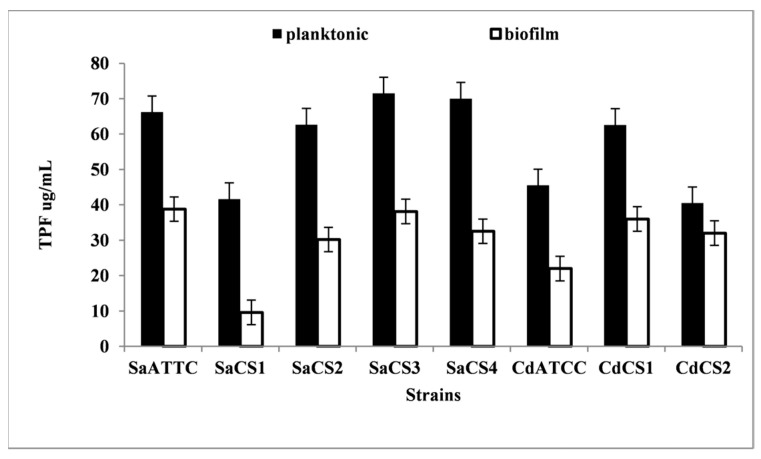
Metabolic activity (TTC reduction) in planktonic cultures and biofilms of *S. aureus* and *C. dubliniensis*, with columns representing triplicate determinations’ average value.

*Planktonic Growth* vs. *Attached Growth.* Reports indicate significant differences in biofilm formation among species [42]. A preliminary test was conducted to assess both species’ general ability to develop biofilms under experimental conditions and their attachment capacity to test tube walls. Biofilm growth was notable in most cases (Figure 3).

**Figure 4:**
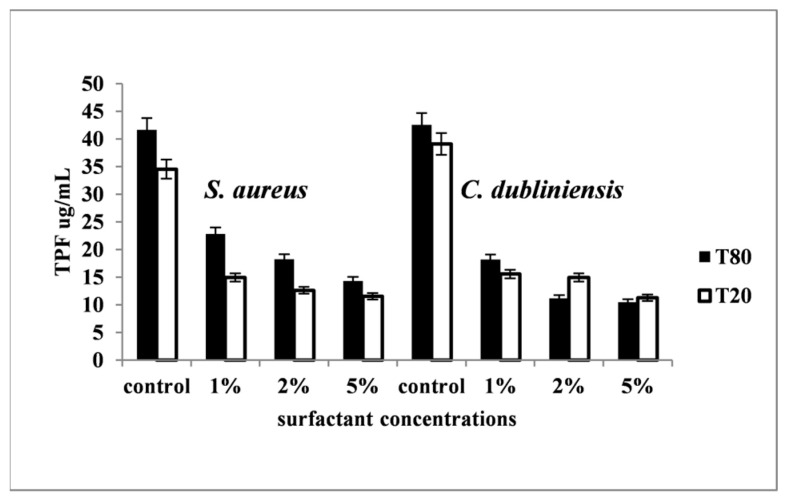
*S. aureus* and *C. dubliniensis* biofilm development at different T20 and T80 concentrations. *S. aureus* SaCS2, *S. aureus* SaCS3, *S. aureus* SaCS4, *C. dubliniensis* CdCS1, and *C. dubliniensis* CdCS1 were used for the experiments. Each column represents the average value of three *S. aureus* strains’ individual readings or two *C. dubliniensis* strains.

*Staphylococcus* strains *S. aureus* ATCC and *S. aureus* SaCS3 exhibited the most consistent biofilm development. However, testing focused on three clinical strains, namely, *S. aureus* SaCS2, *S. aureus* SaCS3, and *S. aureus* SaCS4, due to their MRSA status and multiple antibiotic resistances (Table 1), which is, thus, of higher medical importance. Both clinical strains of *C. dubliniensis* demonstrated significant biofilm-producing capabilities (Figure 3) and were retained for subsequent surfactant effect experiments. A relatively weak correlation was noted (Figure 3) in the TPF production by planktonic cells and *C. dubliniensis* biofilms (r = 0.55), suggesting the slower metabolic activity of cells within the biofilm matrix. Conversely, a positive (Figure 4) and significant relationship existed between planktonic cells and biofilms developed by *S. aureus* (r = 0.95).

*Surfactants’ Effect on Attached Growth*. Surfactants’ impact on biofilm development was tested against T20, T80, and SDS, at concentrations of 1%, 2%, and 5%, and 0.1%, 0.2%, and 0.5%, respectively. Researchers have frequently employed the reduction of tetrazolium salts to gauge the metabolic activity of microbial biofilms across various experimental contexts [39,40,43,44,45,46,47]. This technique has revealed a consistent correlation between metabolic activity and the reduction of tetrazolium salts.

**Figure 5:**
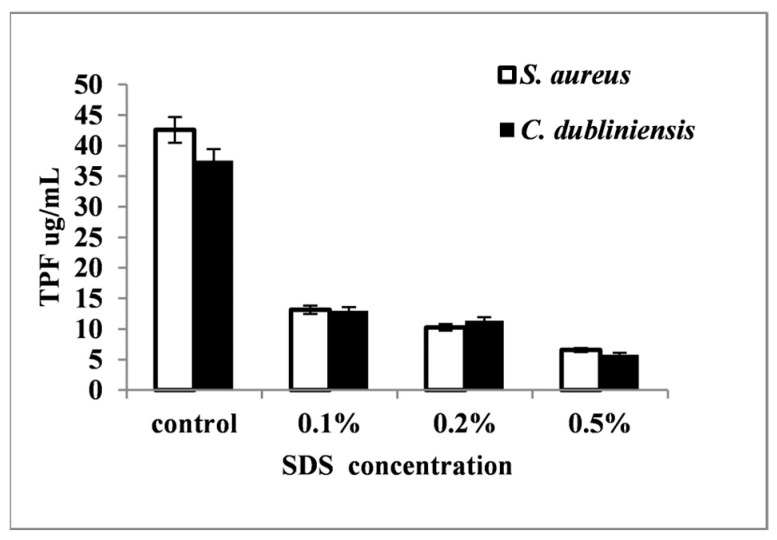
*S. aureus* and *C. dubliniensis* biofilm development at different SDS concentrations, with columns representing the average values from three *S. aureus* strains and two *C. dubliniensis* strains. Biofilm development assessment was based on TTC reduction in experimental variants vs controls.

Observations indicated decreased biofilm development in experimental variants containing surfactants at different concentrations compared to controls (*p* < 0.05). All compounds exhibited varying degrees of biofilm formation inhibition in both species (Figure 4). The biofilm development reduction at varying surfactant concentrations was evaluated based on the metabolic activity (TTC reduction) ratio in controls vs. experimental variants. Moreover, the increased surfactant concentration led to more efficient biofilm inhibition, with the most significant effect observed at 5% (T20, T80) and 0.5% (SDS) (*p* < 0.05). On average, SDS was the most active inhibitor of biofilm development (*p* < 0.05) (Figure 5), while T20 and T80 exhibited weaker and nearly equal effects (Figure 4).

Surfactants’ effects varied based on type and concentration: (i) SDS (0.5%) led to an 85.55% decrease in *S aureus* and *C. dubliniensis* biofilm colonization and development; (ii) T20 (5%) reduced *S. aureus* biofilm development by 75.36% and *C. dubliniensis* by 71.18%; and (iii) T80 (5%) exhibited slightly lower biofilm inhibition, ranging from 65.64% (*S. aureus*) to 66.68% (*C. dubliniensis*) compared to controls.

## 4. Discussion

**Figure 6:**
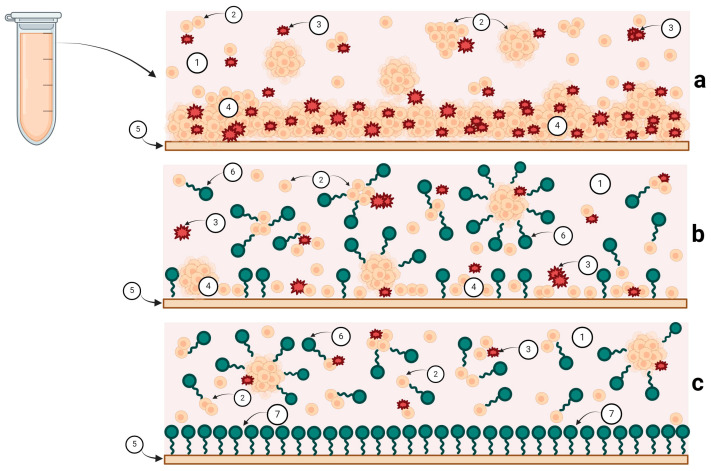
The in vitro impact of surfactants on microbial biofilm development, assessed through TTC reduction. In our experiments, the viability of biofilms was assessed by employing the TTC reduction method, facilitating a simultaneous evaluation of the attachment and biofilm production capabilities of the two species. Surfactants interact with both the cells and the properties of the attachment substrate, hindering optimal cell attachment. The anti-biofilm efficacy was dependent upon both the type and concentration of the surfactants, with the ionic surfactant exhibiting heightened impact even at concentrations ten-fold lower. (**a**) Biofilm growth in the absence of surfactants. (**b**) Intermediate biofilm progression at low surfactant concentrations. (**c**) Inhibition of microbial biofilm growth at high surfactant concentrations. 1. Liquid medium containing cells in suspension; 2. Planktonic microbial cells; 3. Triphenylformazan (TPF) crystals; 4. Cells forming mature biofilms attached to the substrate; 5. Substrate for cell attachment; 6. Surfactant molecules; 7. Surfactant adsorption on the attachment surface alters its hydrophobic properties, hindering microbial cell attachment and biofilm development.

*Substrate Adhesion in Microbial Biofilm Development.* Adhesion to substrates represents the initial critical stage in microbial biofilm development. At the microscale, adhesion is governed by van der Waals, electrostatic, and hydrophobic interactions [48]. The combination of these forces, along with the dominant tendency at a given moment, controls either the cell attraction or repulsion by the substrate [48]. These forces are dynamic and vary depending on the characteristics of the cell surface, chemical composition of the attachment surface, and liquid environment properties where these interactions occur [48,49]. Physical properties of attachment surfaces, such as “micropatterning” at the micrometric scale [49], play an essential role in microbial cell adhesion and the subsequent biofilm development, as this modulates substrate hydrophobicity. A critical condition for cell attachment is the substrate’s hydrophobicity level. The efficacy of the cell attachment to substrates also relies on cell type and the molecular composition of their external surface. Surfactants, amphiphilic molecules, can interfere with both microbial cells [50] and the physical properties of attachment substrates [51]. Surfactants induce changes in cell surface architecture [52,53], including extracellular polymeric substances (EPSs) involved in substrate binding. They also alter the EPS viscoelasticity, disrupting the normal adsorption and attachment process [54,55,56,57]. Surfactants are adsorbed by bacteria, leading to conformational changes in proteins, lipids, and polysaccharides, such as the denaturation or loss of specific functions [53,58,59,60]. The outcomes of these interactions vary depending on the surfactants’ molecular structure and concentration. Ionic surfactants can readily interact with charged molecules, typically amino acids with negatively charged side chains [61] or with lipids to form micelles [62] or vesicles [63] in aqueous environments, resulting in decreased bacterial hydrophobicity, making cells incapable of adhering to surfaces and forming biofilms. Surfactants alter the attachment surface’s hydrophobicity, rendering cells incapable of attachment and biofilm formation (Figure 6). Moreover, surfactants also affect cell properties, hindering surface adsorption. Surface cell adhesion decreases progressively with increasing surfactant concentrations (Figure 6). The concentration exhibiting the maximum inhibitory effect on biofilm formation, in both *Staphylococcus* and *Candida*, depends on the surfactant type, primarily supporting the concept of a physicochemical interaction rather than specific physiological changes in the cells.

Under laboratory conditions, cell attachment depends on substrate properties such as surface hydrophobicity [64,65], culture medium composition, and aerobic conditions [42,66]. A higher planktonic growth of *Candida* is observed on media with a high carbohydrate content [67]. Additionally, the culture medium’s pH value can regulate biofilm formation [68]. *C. dubliniensis* exhibits a robust biofilm-producing capacity [69]. Various chemical compounds, including eugenol [70], fluconazole [71], and unsaturated fatty acids [72], theoretically influence biofilm formation in this species. It has been observed that fatty acids inhibit biofilm development in C. *dubliniensis* to a greater extent than in *C. albicans* [31], suggesting that other fatty acids and likely their esters may prevent planktonic cell adhesion to specific substrates and the subsequent mature *Candida* biofilm formation. Biofilm production by *Staphylococcus aureus* is enhanced by the presence of plasma proteins [73]. Another crucial factor is the intrinsic ability to grow as a biofilm, which varies among species or even strains of the same species [42]. Our experimental findings on surfactant effects align more or less with those of other authors. Unlike Tween, which often stimulates bacterial species’ growth at low concentrations [21], SDS exhibits moderate cidal activity. Apart from biofilm inhibition, SDS could also affect the viability of planktonic microbial cells, reducing their numbers. Numerous factors can influence adhesion and biofilm formation. Ueda et al. [74] reported T80’s inhibitory effect at 0.5% on biofilm development in *Staphylococcus* on a plastic substrate. The effect stemmed from bacterial adhesion inhibition rather than disrupting already formed biofilms, suggesting that T80 may act in the initial phases of biofilm formation. Nielsen et al. [21] argued that T80 at 0.1% stimulated *Staphylococcus* growth after biofilm maturation, whereas, at higher concentrations, like in our experiment, T80 had an inhibitory effect. SDS inhibited growth and biofilm formation in *Candida albicans* [75], attributed to both surfactant and cidal properties. SDS’s inhibitory effect was evident on biofilm development in *Pseudomonas aeruginosa*, while T20 and T80 minimally influenced the biofilm [76,77]. These observations were made on mature biofilms with established structures. In our experiments, *Candida* and *Staphylococcus* were inoculated in media containing surfactants before biofilm establishment. Thus, the recorded inhibition of biofilm development was a direct consequence of changes in normal cell–substrate interactions induced by the surfactant presence. Due to their ability to enhance the solubility of hydrophobic molecules with antimicrobial properties, permeabilize cell membranes, and effectively disrupt micro-organism adhesion to surfaces, surfactants, particularly those with a reduced toxicity, could be employed in various combinations to enhance the efficacy of antimicrobials or optimize nosocomial infection control. Recent studies have focused on finding effective strategies to combat microbial biofilms, including the exploration of (i) natural compounds [78], (ii) antimicrobial nanomaterials and nanoformulations [79], and (iii) antibiofilm coatings of indwelling medical devices [80]. In this context, surfactants could significantly enhance the efficacy of pathogen control, particularly on medical instruments, by integrating them into surface coatings. Furthermore, the antibiofilm efficacy of surfactants should be evaluated against a broader spectrum of micro-organisms, such as *Pseudomonas aeruginosa*, a prolific biofilm producer and a causative agent of recalcitrant infections. Additionally, research efforts should expand to encompass a wider array of surfactant-like molecules to identify the most potent compounds and optimal combinations for enhanced antimicrobial efficacy.

## 5. Conclusions

In conclusion, surfactants (T20, T80, and SDS) affected biofilm growth in *Candida* and *Staphylococcus* in a concentration-dependent manner. Biofilm development inhibition likely resulted from altered substrate physical properties, preventing microbial cell attachment and the subsequent biofilm formation. SDS exhibited the most efficient inhibitory effect, reducing biofilm development by 85.55% in both *S. aureus* and *C. dubliniensis* compared to controls at a concentration of 0.5%. T20 and T80 suppressed biofilm formation only at high concentrations (5%), resulting in a decrease of 2/3 to 3/4 of the value recorded in experimental variants without surfactants. The species’ individual responses demonstrated a moderate variability in response to the surfactant presence, with no significant differences between *Staphylococcus* and *Candida*.

## Figures and Tables

**Table 1:** *Candida* and *Staphylococcus* strains utilized in this study alongside their respective attributes.

Crt. No.	Strain	Observation	Abbreviation
1	*Candida dubliniensis* ATCC MYA-577	Reference strain	CdATTC
2	*Candida dubliniensis* 1	Clinical strain, isolated from oral infection, resistant to fluconazole	CdCS1
3	*Candida dubliniensis* 2	Clinical strain, isolated from oral infection, resistant to fluconazole, ketoconazole	CdCS2
4	*Staphylococcus aureus* ATCC 25923	Reference strain	SaATTC
5	*Staphylococcus* 1	Clinical strain isolated from skin infection (SI), methicillin-resistant *Staphylococcus aureus* (MRSA) resistant to penicillin, ceftarolin, gentamicin, amikacin, kanamycin, azithromicin, erythromycin, tetracyclin, doxyciclin, ciprofloxacin, levofloxacin, clindamycin, trimethoprim-sulfamethoxazole	SaCS1
6	*Staphylococcus* 2	Clinical strain isolated from skin infection (SI), MRSA, resistant to penicillin, ceftarolin, azithromicin, erythromycin, tetracyclin, doxyciclin, trimethoprim-sulfamethoxazole	SaCS2
7	*Staphylococcus* 3	Clinical strain isolated from skin infection (SI), MRSA, resistant to penicillin, tetracyclin, doxyciclin, ciprofloxacin, levofloxacin, clindamycin, trimethoprim-sulfamethoxazole	SaCS3
8	*Staphylococcus* 4	Clinical strain isolated from skin infection (SI), MRSA, resistant to penicillin, amikacin, kanamycin, azithromicin, erythromycin, tetracyclin, doxyciclin, trimethoprim-sulfamethoxazole	SaCS4

## Data Availability

All data generated or analyzed during this study are included in this published article.
